# Supplementary material for: Molecular Quantum Computations on a Protein
Source: J Chem Theory Comput. 2026 Jun 4;22(12):6041–56. doi: 10.1021/acs.jctc.6c00364 (PMC13296500; doi:10.1021/acs.jctc.6c00364)
Supplement: Supplementary file 1 [file ct6c00364_si_001.pdf]

Molecular Quantum Computations on a Protein

Akhil Shajan,[†] Danil Kaliakin,[†] Fangchun Liang,[†] Thaddeus Pellegrini,[‡] Hakan Doga,[‡] Subhamoy Bhowmik,[†,¶] Susanta Das,[†] Antonio Mezzacapo,[‡] Mario Motta,[‡] and Kenneth M. Merz Jr.[*,†,¶]

†Center for Computational Life Sciences, Lerner Research Institute, The Cleveland Clinic, Cleveland, Ohio 44106, United States

‡IBM Quantum, IBM T.J. Watson Research Center, Yorktown Heights, NY 10598, United States

¶*Department of Chemistry, Michigan State University, East Lansing, Michigan 48824, United States*

E-mail: kmerz1@gmail.com

Supporting Information

Choice of interacting bath expansion threshold

We started the benchmark of numerical value for threshold η with $\eta = 1 \cdot 10^{-6}$. We used the relative energy (ΔE) between folded and unfolded conformers of Trp-cage as the metric defining the sufficiency of the η value. Here we targeted the low deviation between the relative energies predicted with EWF calculations and unfragmented calculations. As shown in Table S1 at $\eta = 1 \cdot 10^{-6}$ the prediction of relative energy by EWF-CCSD deviates from relative energy calculated with unfragmented DLNO-CCSD by 3.4 kcal/mol. The prediction of relative energy by EWF-MP2 deviates from relative energy calculated with unfragmented RI-MP2 by 1.9 kcal/mol.

Table S1: Effect of interacting bath expansion threshold (η). First column shows the used method with inclusion of η for corresponding EWF calculations. Second column demonstrates the relative energies (ΔE) between folded and unfolded conformers of Trp-cage in kcal/mol. Third column denotes maximum number of MOs in resulting EWF clusters ($EWF_{max\text{-}MOs}$) at given value of η. Fourth column shows maximum number of qubits ($EWF_{max\text{-}SQD\text{-}Qubits}$) that would be required to be used in SQD simulations of largest EWF clusters at given value of η.

Method	ΔE	$EWF_{max\text{-}MOs}$	$EWF_{max\text{-}SQD\text{-}Qubits}$
EWF-CCSD(η= $1 \cdot 10^{-6}$)	-48.66	46	96
EWF-CCSD(η= $1 \cdot 10^{-5}$)	-47.70	33	70
EWF-MP2(η= $1 \cdot 10^{-6}$)	-61.49	46	96
EWF-MP2(η= $1 \cdot 10^{-5}$)	-59.61	33	70
DLPNO-CCSD	-52.05		
RI-MP2	-63.42		

The maximum qubit count (96 qubits) observed at $\eta = 1 \cdot 10^{-6}$ is beyond the qubit range that was shown with SQD and ext-SQD methods to date.[1-4] Since the exploration of larger qubit counts in individual SQD runs was outside the scope of the present study we adjusted the η threshold as $\eta = 1 \cdot 10^{-5}$ resulting in the maximum qubit count of 70 which is within the range that was shown to be optimal for SQD and ext-SQD simulations in previous studies.[1-4] To elucidate whether $\eta = 1 \cdot 10^{-5}$ results in the reasonably accurate prediction of relative energies of Trp-cage conformers we repeated the EWF-CCSD and EWF-MP2 calculations at $\eta = 1 \cdot 10^{-5}$. At $\eta = 1 \cdot 10^{-5}$ the prediction

of relative energy by EWF-CCSD deviates from relative energy calculated with unfragmented DLNO-CCSD by 4.4 kcal/mol. The prediction of relative energy by EWF-MP2 deviates from relative energy calculated with unfragmented RI-MP2 by 3.8 kcal/mol. Hence, the adjustment of η threshold allowed us to achieve the manageable qubit count without significant deprecation of accuracy in simulations of relative energy between folded and unfolded conformers of Trp-cage.

Visualization of atoms producing the EWF clusters with largest number of MOs

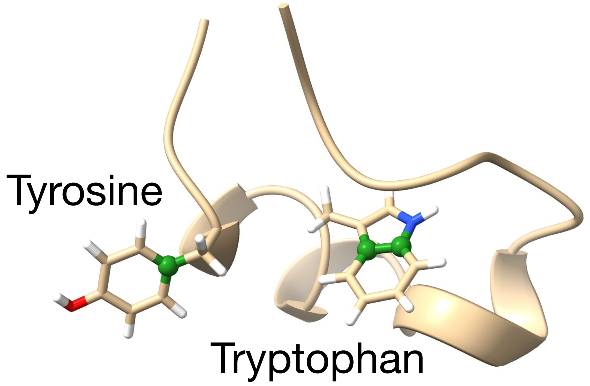

Figure S1. Visualization of atoms in folded conformer of Trp-cage corresponding to EWF clusters with 33 MOs. Only atoms of in tryptophan and tyrosine are shown for clarity. Light brown, light grey, blue, and red atoms correspond to carbon, hydrogen, nitrogen, and oxygen atoms, respectively. Atoms marked in green signify the carbon atoms of benzene rings in tryptophan and tyrosine which correspond to largest EWF clusters with 33 MOs.

Average classical compute time for SQD and ext-SQD simulations of EWF clusters

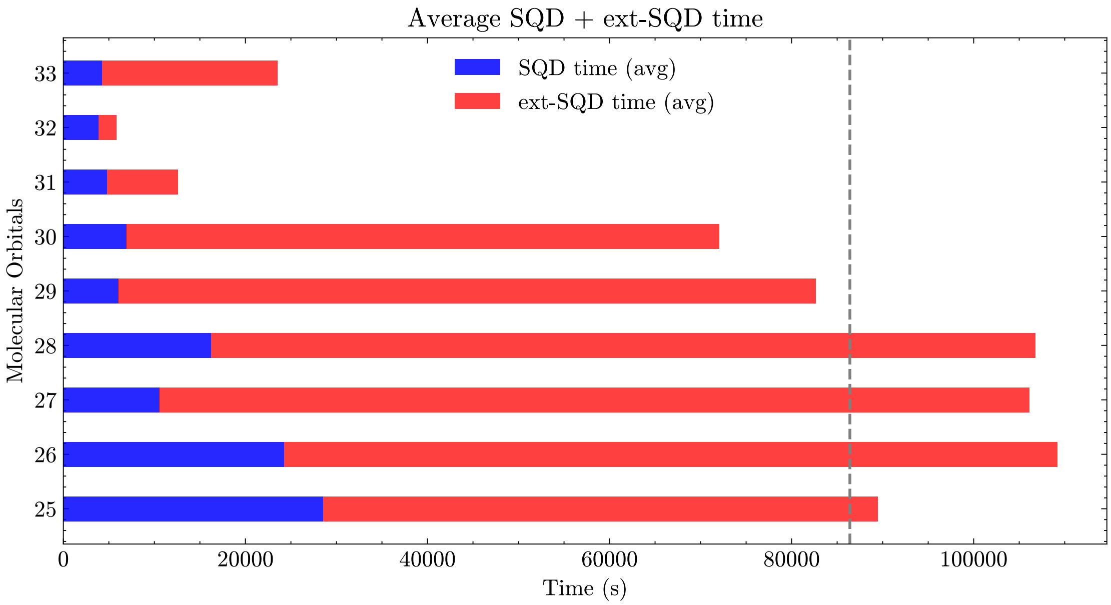

Figure S2: Plot shows the average classical compute time (in seconds) of SQD and ext-SQD Simulations for EWF clusters ranging 25-33 MOs. The vertical dashed line marks the 24 hour total time. Since the values are comparable between folded and unfolded conformers, the plot shows the average times for the folded conformer only.

Structures of folded and unfolded conformers of Trp-cage

The structures of folded and unfolded conformers of Trp-cage utilized in this study are provided in the supplied xyz files.

References

(1) Robledo-Moreno, J.; Motta, M.; Haas, H.; Javadi-Abhari, A.; Jurcevic, P.; Kirby, W.; Martiel, S.; Sharma, K.; Sharma, S.; Shirakawa, T. et al. Chemistry beyond the scale of exact diagonalization on a quantum-centric supercomputer. *Science Advances* **2025**, *11*, eadu9991.

(2) Shirakawa, T.; Robledo-Moreno, J.; Itoko, T.; Tripathi, V.; Ueda, K.; Kawashima, Y.; Broers, L.; Kirby, W.; Pathak, H.; Paik, H. et al. Closed-loop calculations of electronic structure on a

quantum processor and a classical supercomputer at full scale. *arXiv preprint* **2025**, arXiv:2511.00224
(3) Barison, S.; Moreno, J. R.; Motta, M. Quantum-centric computation of molecular excited states with extended sample-based quantum diagonalization. *Quantum Science and Technology* **2025**, *10*, 025034.
(4) Barroca, M. A.; Gujarati, T.; Sharma, V.; Neumann Barros Ferreira, R.; Na, Y.-H.; Giammona, M.; Mezzacapo, A.; Wunsch, B.; Steiner, M. Surface reaction simulations for battery materials through sample-based quantum diagonalization and local embedding. *arXiv preprint* **2025**, arXiv: arXiv:2503.10923
